# Setting the optimal positive end-expiratory pressure: a narrative review

**DOI:** 10.3389/fvets.2023.1083290

**Published:** 2023-07-19

**Authors:** Kristin M. Zersen

**Affiliations:** Department of Clinical Sciences, Colorado State University, Fort Collins, CO, United States

**Keywords:** mechanical ventilalion, PV loops, positive end expiratory pressure (PEEP), driving pressure, compliance, stress index, transpulmonary pressure, electrical impedance tomograghy

## Abstract

The primary goals of positive end-expiratory pressure (PEEP) are to restore functional residual capacity through recruitment and prevention of alveolar collapse. Through these mechanisms, PEEP improves arterial oxygenation and may reduce the risk of ventilator-induced lung injury (VILI). Because of the many potential negative effects associated with the use of PEEP, much research has concentrated on determining the optimal PEEP setting. Arterial oxygenation targets and pressure-volume loops have been utilized to set the optimal PEEP for decades. Several other techniques have been suggested, including the use of PEEP tables, compliance, driving pressure (DP), stress index (SI), transpulmonary pressures, imaging, and electrical impedance tomography. Each of these techniques has its own benefits and limitations and there is currently not one technique that is recommended above all others.

## Peep definition: what is the benefit of its use?

Positive end-expiratory pressure (PEEP) has been used during mechanical ventilation for decades, and it was first described by Ashbaugh and colleagues when they noted the benefits of PEEP in patients with acute respiratory distress syndrome (ARDS) (1, 2). Most of the studies evaluating PEEP in the human literature are performed in patients with ARDS. While this is a specific patient population, results of this research are commonly applied across patients with multiple pulmonary diseases.

PEEP ensures that the pressure in the alveoli is higher than atmospheric pressure, and this creates a positive baseline pressure (3). PEEP can be further defined as extrinsic or intrinsic. This review will focus on extrinsic PEEP, which is a ventilator setting that is controlled by the operator. Intrinsic PEEP, or auto-PEEP, is most commonly associated with inadequate expiratory times, collapse of small airways, or increased airway resistance. This may be due to a small endotracheal tube, bronchospasm, or accumulation of secretions (4).

Functional residual capacity (FRC) is the volume of air remaining in the lungs after a normal expiration and is an important oxygen reserve that allows for continued gas exchange (5). Reduced FRC results in less alveolar tension pulling airways open, and subsequently, airway narrowing or collapse and increased airway resistance (5). FRC is decreased by many factors important to mechanically ventilated patients including lung compliance, patient position, and anesthesia/drugs. Mechanically ventilated patients with pulmonary disease, including ARDS, will have decreased lung compliance which contributes to a decrease in FRC. Additionally, mechanically ventilated patients are maintained in a supine position which also decrease FRC. Finally, anesthetic drugs and sedatives decrease the tone of respiratory muscles, further contributing to a decrease in FRC.

The primary goals of PEEP are to restore FRC through: (1) recruitment of alveoli, which decreases intrapulmonary shunting, and (2) prevention of alveolar collapse, which may occur due to surfactant impairment, increased lung weight, and chest wall recoil (3). Clinically, the goal is to improve arterial oxygenation. When PEEP is applied, the end expiratory lung volume (EELV) increases, which is predominately due to the recruitment of collapsed alveoli (6). An increased EELV leads to decreased lung strain and improved compliance, ultimately contributing to a decrease in DP (3).

Through these mechanisms, PEEP improves arterial oxygenation and may reduce the risk of ventilator-induced lung injury (VILI) by reducing atelectrauma resulting from the cyclic opening and closing of alveoli, preventing alveolar flooding, and reducing lung heterogeneity (3). Alveolar recruitment also reduces lung strain and improves lung compliance in both humans and dogs (6, 7). In a study of healthy, mechanically ventilated dogs, the addition of 5 mmHg PEEP improved compliance and decreased DP (7). In addition, PEEP reduced global and regional dynamic lung strain, but it also increased static strain (7). Dynamic lung strain has been shown to be more injurious than static lung strain, so the addition of PEEP may be considered beneficial based on this research (7).

## Negative effects from PEEP

PEEP can have significant negative cardiovascular and pulmonary effects. PEEP contributes to decreased cardiac output (CO) through two primary mechanisms. First, PEEP increases intrathoracic pressure, which contributes to increasing right atrial pressure and decreased venous return (6). Second, PEEP may increase pulmonary vascular resistance, which results in increased right ventricular afterload (6).

As previously mentioned, EELV increases when PEEP is applied, predominately due to the recruitment of collapsed alveoli. However, in low compliance lung regions or in alveoli that are already recruited, the increased EELV may also contribute to alveolar overdistention and a decrease in dynamic strain (3). Overdistention may lead to alveolar inflammation, injury, and increased lung stress. In addition, if alveolar pressure is greater than pulmonary capillary pressure, capillaries may be occluded and lead to increased alveolar dead space (3).

The risks associated with PEEP are amplified because of the heterogenous nature of lung injuries. Inevitably, there will be some regions of lung that are relatively normal, some regions of lung that will be recruited with the application of PEEP, and some regions of lung that are so diseased that they are not recruitable. The clinician cannot select which lung regions to target, so PEEP will be applied to all lung regions, leading to overdistention of some alveoli and recruitment of other alveoli.

The addition of increasing PEEP during mechanical ventilation has been shown to increase anatomic and alveolar dead space in a pig model of ARDS (8). Dead space includes gas that does not participate in gas exchange and is often referred to as wasted ventilation (9). However, a study of healthy horses undergoing laparotomy, showed that adding an end-inspiratory pause reduced the alveolar dead space and physiologic dead space ventilation associated with PEEP (9).

## Which patients will benefit from PEEP?

The benefits of PEEP are well-described in people with ARDS, where PEEP is used to prevent VILI. While a comprehensive review of VILI is outside the scope of this review, VILI is generally thought to be due to volutrauma, biotrauma, and atelectrauma. Because of this, a lung protective strategy including low tidal-volume and the use of PEEP has been suggested in these patients.

Because of the many potential negative effects associated with the use of PEEP, much research has concentrated on determining the optimal PEEP setting. Human studies have shown that using higher versus lower levels of PEEP did not significantly improve survival (1, 10, 11). However, additional analysis of these studies documented reduced mortality in patients with moderate-to-severe ARDS receiving higher PEEP (12). There is not a single, consistent definition of higher and lower PEEP in the literature, however, in one meta-analysis the mean PEEP setting in the higher PEEP group was 15.3 cm H_2_O and the lower PEEP group was 9.0 cm H_2_O (12). However, thoracic compliance is different in dogs and cats compared to humans, so these values may not be directly applicable. One theory regarding the differences in patient responses is the difference in lung recruitability. If PEEP can improve alveolar recruitment, it will be beneficial. But, if PEEP is applied without recruitment, serious complications and even worse outcomes are likely. To support this theory, one study showed that patients who responded to increased PEEP by improved oxygenation had a lower risk of death (13). Unfortunately, there are limited techniques available to assess the potential of alveolar recruitment bedside. A technique for assessing response to a recruitment maneuver using point-of-care ultrasound (POCUS) has been described in humans (14). The technique involves visualizing the most dependent zone of atelectasis with POCUS and monitoring for resolution of consolidation and re-aeration (14). Another technique for assessing lung recruitability is measurement of the recruitment-to-inflation ratio (R/I ratio). A technique for measuring the R/I ratio has been described previously and studies have shown that the R/I ratio can help characterize recruitability bedside (15). While oxygenation is not a perfect marker of alveolar recruitment, it is one of the most commonly used in clinical settings at this time.

## Techniques for setting the optimal PEEP

The first study that aimed to define optimal PEEP was published by Suter and colleagues in 1975. They defined optimal PEEP as the value associated with the best oxygen delivery and dead space reduction, and this was based on assessment of arterial oxygenation, hemodynamics, and respiratory mechanics (16). Arterial oxygenation targets remain the most widely used technique for setting PEEP in practice. In the 1980s, the use of pressure-volume (PV) loops to set optimal PEEP was first introduced. Matamis and colleagues recommended setting PEEP based on the lower inflection point on the PV loop (17). This technique is still used in clinical practice today. In the 2000s, the focus transitioned from arterial oxygenation to lung protection. From the 2000s to present day, several other techniques for setting the optimal PEEP have been suggested, including the use of PEEP tables, compliance, DP, SI, transpulmonary pressures, imaging, and electrical impedance tomography.

### Recruitment maneuvers

A recruitment maneuver (RM) is a technique used to recruit collapsed alveoli and involves temporarily increasing the pressure delivered via mechanical ventilation. The amount of pressure needed to recruit collapsed alveoli is highly variable depending on the severity of pulmonary disease and patient demographics. In one veterinary study of mechanically ventilated healthy beagles, a recruitment airway pressure of 15 cmH_2_O was recommended as it reduced the amount of hypoaerated lung without overdistending the lung (18). It is unknown if this recruitment airway pressure applies to dogs with pulmonary disease.

Once the threshold opening pressure (TOP) of each alveoli is reached, the alveoli will open. The TOP will vary throughout the lung, so some alveoli will be temporarily overdistended in an effort to reach the TOP for as many alveoli as possible. The potential risks associated with the use of an RM include volutrauma, barotrauma, alveolar capillary injury, and decreased CO due to decreased venous return (19, 20).

There are many different techniques for performing a RM described in the veterinary literature, including but not limited to:

- Inspiratory hold for 30 s at 15 cmH_2_O airway pressure (18).
- Increase PEEP and end inspiratory pressure (EIP) by 5 cmH_2_O every 1 min until PEEP of 15 cmH_2_O is reached, at which time EIP is increased to 30 cmH_2_O, for one minute (21).
- Increase airway pressure to 40 cmH_2_O for 20 s (22).
- Continuous positive airway pressure of 40 cmH_2_O for 20 s (23).
- PEEP applied in steps of 5, 10, 15, and 20 cmH_2_O every 5 breaths until a plateau pressure of 40 cmH_2_O is reached and maintained for one minute (23).

While the above RMs have been described in the veterinary literature, it is important to note that an airway pressure of 15 cmH_2_O may not be considered a RM in human medicine. Additionally, a continuous positive airway pressure of 40 cmH_2_O and incremental increases in PEEP to a plateau pressure of 40 cmH_2_O, are not recommended in human ARDS patients.

There is not sufficient evidence to recommend the routine use of RMs. If a RM is used, a stepwise RM is recommended over a sustained inflation RM (24). Once the RM is complete, PEEP should be adjusted to maintain the recruitment and prevent de-recruitment.

### PEEP/FiO_2_ tables

The ARDS Network first published a PEEP/FiO_2_ table in 2000 when they documented improved survival in patients ventilated with lower tidal volumes as opposed to higher tidal volumes (1). Using this table, commonly referred to as the lower PEEP/FIO_2_ table (see Table 1), patients undergoing low tidal volume ventilation have been shown to have improved survival (25). A second table using higher PEEP values, commonly referred to as the higher PEEP/FiO_2_ table (see Table 2), has also been published (26). Improved oxygenation has been demonstrated with the use of the higher PEEP/FiO_2_ table; however, there has been no difference in mortality between the lower and higher PEEP/FiO_2_ tables in multiple studies (11, 26).

**Table 1:** Lower PEEP/FiO_2_ table.

FiO_2_	0.3	0.4	0.4	0.5	0.5	0.6	0.7	0.7	0.7	0.8	0.9	0.9	0.9	1.0
PEEP	5	5	8	8	10	10	10	12	14	14	14	16	18	18–24

Table to guide PEEP settings using incremental steps in FiO2 and PEEP to maintain a PaO_2_ of 55–80 mmHg. This is commonly referred to as the lower PEEP/FiO_2_ table. FiO_2,_ fraction of inspired oxygen; PaO_2_, partial pressure of arterial oxygen. Adapted from: Ref. (1).

**Table 2:** Higher PEEP/FiO_2_ table.

FiO_2_	0.3	0.3	0.3	0.3	0.3	0.4	0.4	0.5	0.5	0.5–0.8	0.8	0.9	1.0
PEEP	5	8	10	12	14	14	16	16	18	20	22	22	22–24

Table to guide PEEP settings using incremental steps in FiO_2_ and PEEP to maintain a PaO_2_ of 55–80 mmHg. This is commonly referred to as the higher PEEP/FiO_2_ table. FiO_2_, fraction of inspired oxygen; PaO_2_, partial pressure of arterial oxygen. Adapted from: Ref. (26).

Using these tables involves adjusting PEEP and FiO_2_, such that PEEP targets must be met before FiO_2_ is increased further. Adjustments are made based on the table until oxygenation goals of SpO_2_ 88–95% and/or PaO_2_ 55–80 mmHg are met. The tables are easy to use in clinical practice and increase average PEEP levels across all patient populations (6). However, the goal of these tables is to improve oxygenation, and as discussed previously, PEEP will not improve oxygenation in patients if they do not have capacity for lung recruitment.

### Pressure volume loops

**Figure 1:**
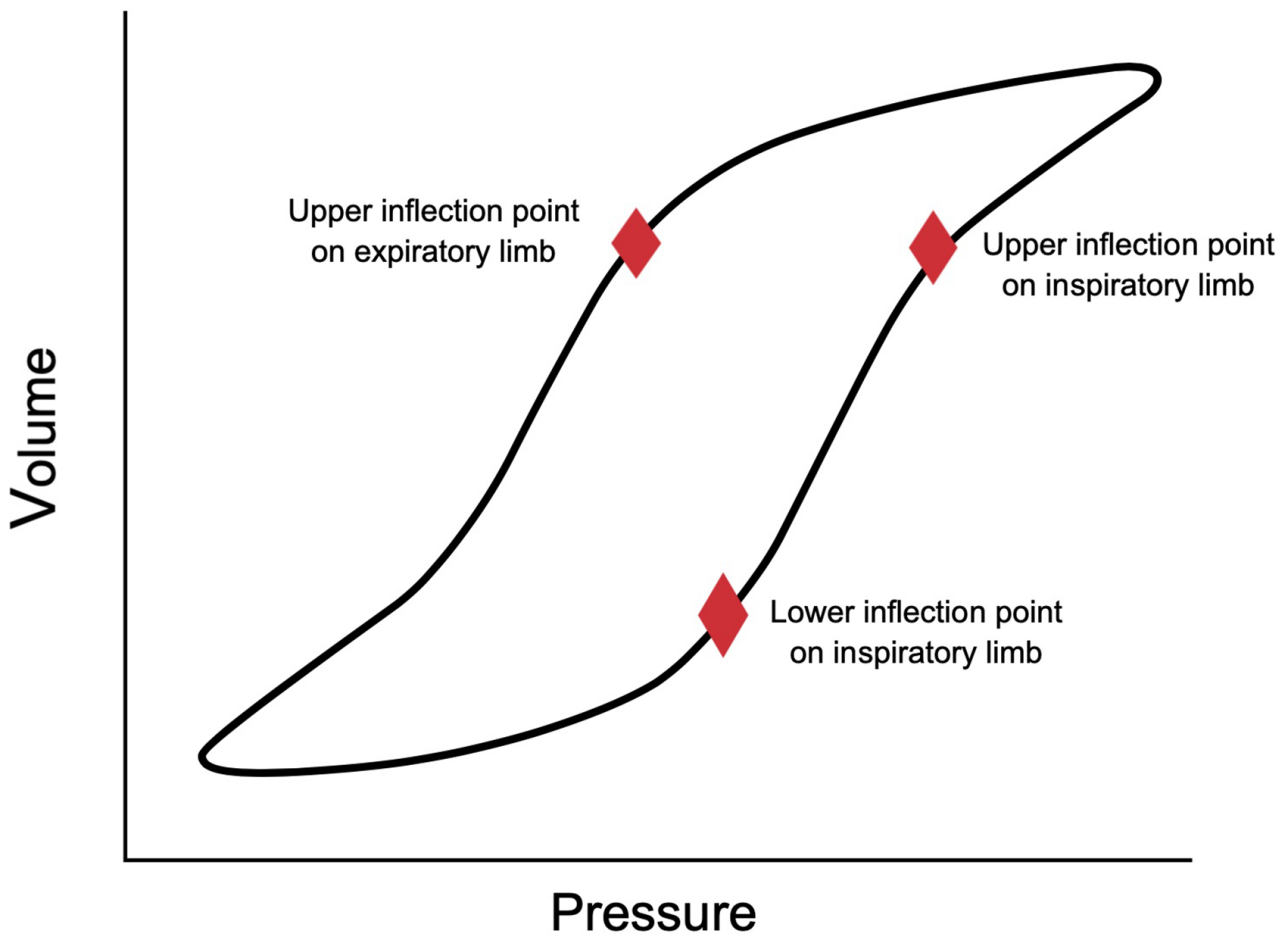
Pressure-volume loop with labeled inflection points.

PV loops represent the dynamic interaction of changes in pressure and volume during the inspiratory and expiratory portions of a breath. They are used to assess lung mechanics in mechanically ventilated patients, most commonly to monitor changes in lung compliance and airway resistance. When there is a decrease in lung compliance, the PV loop rotates closer to the x-axis, lying more horizontally (27). When there is an increase in compliance, the PV loop rotates toward the y-axis, lying more vertically (27). Two important inflection points are also described on the static PV loop (Figure 1). The lower inflection point (LIP) is located on the inspiratory limb of the PV loop. This represents the point at which compliance increases significantly, likely due to the recruitment and opening of alveoli (27). The upper inflection point (UIP) on the inspiratory limb has been suggested to be the point at which compliance decreases due to the overdistension of alveoli (27). This decrease in compliance and overdistension of alveoli creates a classic beaking appearance to the PV loop. However, some studies have challenged this theory and suggest that recruitment can continue to occur above the UIP (28, 29).

In 1984, Matamis and colleagues recommended the use of PV loops to set optimal PEEP. They suggested setting PEEP above the LIP on the inspiratory limb of the PV loop to prevent distal airway collapse and to maximize alveolar recruitment (17). This technique of assessing the PV loop to set the optimal PEEP is still used today by many clinicians.

There are two techniques currently recommended for using the PV loop to set PEEP. The first technique involves setting PEEP at 2 cmH_2_o higher than the inspiratory limb LIP (19). It is important to note that it is not always easy to identify the LIP, and in some patients, they do not have a LIP (19). More recently, it has been suggested that PEEP should be set at the UIP of the expiratory limb of the PV loop (19) (Figure 1). This recommendation is based on the fact that de-recruitment is an expiratory phenomenon, so setting PEEP above the expiratory limb UIP would minimize de-recruitment (19). Multiple studies have shown that setting PEEP above the expiratory limb UIP promotes alveolar stability and produces greater alveolar recruitment compared to setting PEEP above the inspiratory limb LIP (30, 31). However, this technique may be more likely to cause volutrauma (32).

Finally, the open lung ventilation strategy suggests setting PEEP above the inspiratory limb LIP and setting tidal volume (TV), so the plateau pressure (P_plat_) is below the UIP (33). This strategy has been shown to improve outcomes in patients with ARDS (33). The primary downside to using PV loops is the ability to acquire a reliable PV loop without artifact (19). Additionally, neuromuscular blockade and breath holds for static assessments are required to obtain adequate PV loops. This is technically challenging which limits its clinical use.

### Compliance

**Figure 2:**
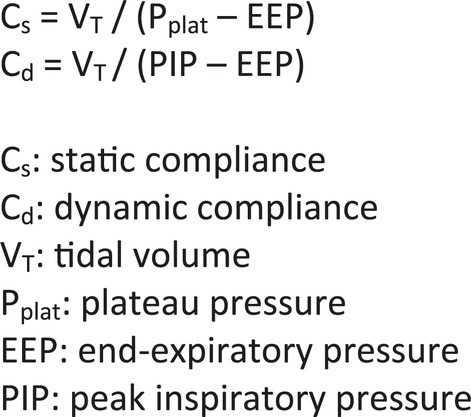
Compliance formulas.

Compliance is defined as the change in lung volume per unit change in pressure (C = Δ V/Δ P) and it describes the distensibility of the lung (3). Compliance is further described as static compliance (C_s_) and dynamic compliance (C_d_). C_s_ is defined as the pulmonary compliance during no-airflow conditions and is measured during an inspiratory hold (34). C_d_ is defined as pulmonary compliance measured during breathing and is influenced by both compliance and resistance (35). Formulas for calculating C_s_ and C_d_ are in Figure 2. The measurement of plateau pressure is required to calculate C_s_ and is obtained using an inspiratory hold technique.

Suter and colleagues were the first group to recommend adjusting PEEP to maximize oxygen delivery, which they determined by measuring CO and arterial oxygen content (CO × arterial oxygen content) (16). In this study, maximum oxygen delivery was achieved at the PEEP associated with the highest C_s_. Since this time, other studies have supported this technique, demonstrating improved organ function and arterial oxygenation when PEEP is titrated to maximize C_s_ (36, 37). There are concerns with using C_s_ to set the optimal PEEP as C_s_ does not always increase after the administration of PEEP, even when there is significant lung recruitment documented with CT (38). It is also important to note that compliance measurements are global estimates and do not take into account regional variations, when we know that alveolar recruitment and overdistension are heterogeneously distributed (39). One veterinary study of healthy dogs found that setting PEEP at C_s_ or C_s_ + 2 cmH_2_O did not result in improved arterial oxygenation or oxygen delivery index (40). Setting PEEP at Cs + 4 cmH_2_O resulted in decreased oxygen delivery index (40).

To implement this technique, an RM should be performed and PEEP should be increased. Then, PEEP should be decreased in a stepwise fashion and C_s_ measured at each change. The PEEP that produces the highest C_s_ is the appropriate PEEP stetting for that patient. A second RM can be performed, followed by setting PEEP at the appropriate setting based on the previous C_s_ measurements (19).

### Driving pressure

DP is calculated as the difference between inspiratory plateau pressure and PEEP, or the ratio of TV to compliance (DP = P_plat_ – PEEP or DP = TV/compliance) (41). In the absence of respiratory effort by the patient, DP represents the pressure above PEEP which is applied to the respiratory system to achieve ventilation (41). It reflects the size of TV relative to aerated lung volume, and therefore, correlates with overall lung strain and pulmonary compliance (6, 41).

DP has been shown to be a strong predictor of lung stress and outcome. ARDS patients with a DP > 7 cmH_2_O have been shown to have an increased risk for mortality (42) and in a more recent study, a DP of >14 cmH_2_O on day 1 had a worse outcome (43). DP has also been associated with lung stress, such that higher DPs have significantly higher lung stress (44). Finally, decreases in DP have been shown to be more strongly associated with lower mortality compared to increases in the PaO_2_/FiO_2_ ratio, confirming that DP was the key variable associated with outcome (45). In a veterinary study of healthy dogs, adding PEEP (5 cmH_2_O) significantly decreased DP (7). To implement this technique, DP should be measured at different levels of PEEP while maintaining a constant tidal volume. If PEEP is raised and DP decreases, this suggests the higher PEEP has resulted in increased compliance and increased alveolar recruitment. If PEEP is raised and DP increases, this suggests the higher PEEP has resulted in decreased compliance and hyperinflation (6).

### Stress index

SI is measured by determining the slope of the airway pressure–time curve during inspiration, based on two timepoints on a dynamic airway pressure scaler (33). The measurement of SI requires volume-controlled ventilation and a constant flow pattern, which keeps alveolar volume and pressure constant (33). Under these conditions, the slope of the airway pressure rise will represent changes in compliance.

SI is most valuable for detecting alveolar recruitment and hyperinflation (46). SI increases, or SI > 1, indicates decreasing compliance, most commonly due to hyperinflation (6). SI decreases or SI < 1 indicates increasing compliance, which may be a marker of alveolar recruitment (6). Using SI to determine optimal PEEP involves setting PEEP to a pressure at which the SI = 1 (6). When SI = 1, it is thought that neither hyperinflation or recruitment is occurring.

In one study, PEEP was set using the low PEEP/FiO_2_ table in one group and SI in a second group. PEEP was lower in all patients in which PEEP was set using SI and was associated with higher compliance, lower PaCO_2_, and no significant difference in PaO_2_/FiO_2_ (47). The clinical utility of measuring SI is limited because of the need for quantitative analysis of the shape of the pressure–time curve with dedicated instruments or specific ventilators. However, a recent study has suggested that SI can be reliably assessed bedside with visual inspection of pressure-time curves. (48). However, a recent study has suggested that SI can be reliably assessed with visual inspection of pressure–time curves (48).

### Transpulmonary pressure

Transpulmonary pressure (P_TP_) is defined as the difference between the airway pressure and the pleural space pressure, and it represents the pressure required to move air through the airways and to overcome elastic recoil (41). When measured at end-inspiration and end-expiration, airway pressures represent alveolar pressures, and P_TP_ represents the stress applied directly to the lung, independent of the chest wall (41). Pleural pressure is most commonly estimated by measuring esophageal pressure using esophageal manometry. Esophageal pressure is most commonly measured using an air or fluid-filled catheter positioned in the distal third of the esophagus. There are multiple techniques for estimating pleural pressure from esophageal pressure measurements, including using the absolute value of esophageal pressure and using the changes in esophageal pressure during tidal insufflation (3).

P_TP_-guided PEEP approaches have been shown to improve oxygenation, increase compliance, and decrease DP (49, 50). However, a more recent study showed no difference in mortality rate or days free of mechanical ventilation when comparing an esophageal pressure-guided PEEP strategy and a PEEP-FiO_2_ strategy (51).

One veterinary study evaluated the specific lung elastance, the P_TP_ at which the lung doubles its FRC, in anesthetized dogs. They determined that the specific lung elastance was 12.7 cmH_2_O, which is similar to the specific lung elastance in humans (52). Additionally, when a specific lung elastance based recruiting airway pressure was applied, aeration of previously poorly and non-aerated lung tissue improved, however, hyperinflation was also observed (52).

If implementing this technique, current human recommendations are to adjust PEEP to ensure a positive end-expiratory P_TP_, usually 0–10 cmH_2_O, which avoids end-expiratory alveolar collapse (41). Talmor and colleagues published a table in which PEEP is set to achieve an end-expiratory P_TP_ based on the FiO_2_ to maintain PaO_2_ of 55–120 mmHg (Table 3).

**Table 3:** Expiratory transpulmonary pressure table.

FiO_2_	0.4	0.5	0.5	0.6	0.6	0.7	0.7	0.8	0.8	0.9	0.9	1.0
P_TP_	0	0	2	2	4	4	6	6	8	8	10	10

Table to guide PEEP setting based on P_TP_. Set PEEP to achieve P_Tp_ based on the FiO_2_ to maintain PaO_2_ of 55–120 mmHg. FiO_2_, fraction of inspired oxygen; P_Tp_, expiratory transpulmonary pressure; PaO_2_: partial pressure of arterial oxygen. Adapted from Ref. (50).

### Imaging

CT has been shown to correlate with alveolar recruitment and de-recruitment, but other studies have shown that lung recruitability and CT scan-derived PEEP were not related (53). Performing repeated CT scans is impractical in the clinical setting, so its use is generally not recommended for guiding PEEP settings.

Lung ultrasound has been proposed to be a more practical method for imaging the lung and has been shown to be effective in evaluating and guiding alveolar recruitment (54). An ultrasound re-aeration score has been published and was shown to be correlated with increases in oxygenation and increases in lung recruitment (55). However, ultrasound cannot be used to assess for lung hyperinflation. Similarly, a technique for assessing response to a recruitment maneuver using POCUS has been described in humans (14). The technique involves determining the lung opening pressure and closing pressure in the most dependent zone of atelectasis. Once this zone is identified with POCUS, the recruitment maneuver is performed and the point at which the consolidation pattern disappears and re-aeration is observed, is defined as the lung opening pressure. If a decremental PEEP trial is used, the closing pressure can be identified as the pressure at which consolidation is first noted. PEEP should then be set 2 cmH_2_O above the closing pressure (14).

### Electrical impedance tomography

Electrical impedance tomography (EIT) is a non-invasive bedside technique which allows real-time visualization of changes in the distribution of ventilation and perfusion. Measuring EIT involves placing several electrodes around the patient’s chest, which measures thoracic impedance to small alternating electrical currents that are applied through electrodes (56). Software analyzes this data and creates an image of the lung depicting ventilation and perfusion, which is displayed on a monitor (56).

There are multiple EIT techniques that have been described for setting the optimal PEEP; however, the originally described technique is still the one most used. Following an RM, the original technique proposes estimating changes in regional lung compliance during a decremental PEEP trial (57). The PEEP should then be set at the point of intersection between the collapse and overdistention percentage curves assessed by EIT (57). Multiple studies comparing EIT-guided PEEP to PV curve-guided PEEP have shown higher compliance, lower DP, and improved survival in the EIT-guided PEEP group (58, 59). There are limitations to the use of EIT, as the only lung evaluated is the lung surround directly by the belt, so consistent belt placement is important for re-evaluation (56).

## Conclusion

Although it is well-accepted that PEEP is a valuable tool to improve oxygenation in mechanically ventilated patients, the use of PEEP has not always been associated with a decrease in mortality, and therefore, it’s importance in mechanical ventilation can be debated. There are many techniques available to set the optimal PEEP, and each has its own benefits and limitations. There is a very diverse population that requires mechanical ventilation, so there will likely never be a technique that is ideal in all patients. When PEEP is decreased, changes can be assessed quickly as PaO_2_ and arterial oxygen saturation reach equilibrium within 5 min (60). However, when PEEP is increased, it may take over 1 h for PaO_2_ and arterial oxygen saturation to reach equilibrium (60). Therefore, it is important to not make additional increases to PEEP rapidly.

There are significant limitations in the veterinary literature regarding the use of PEEP. The publications discussed in this review are limited to research in dogs with normal lungs, so the results cannot necessarily be applied to veterinary patients receiving mechanical ventilation for pulmonary disease. None of the techniques discussed have been validated in veterinary patients outside of animal models of acute lung injury and ARDS. However, the most clinically relevant techniques for setting the optimal PEEP in veterinary patients likely include the use of PEEP/FiO_2_ tables, PV loops, compliance, and DP. Point-of-care lung ultrasound may prove to be a valuable technique, but this will likely require veterinary specific data to be clinically useful.

In the future, validation of these techniques in veterinary patients would be beneficial; however, conducting clinical trials with sufficient patient numbers will continue to be a challenge. Future research in human patients will likely focus on the development of bedside methods to quantify lung recruitability and overdistension, so patients that will benefit from the application of PEEP will be readily identified.

## Author contributions

The author confirms being the sole contributor of this work and has approved it for publication.

## Conflict of interest

The authors declare that the research was conducted in the absence of any commercial or financial relationships that could be construed as a potential conflict of interest.

## Publisher’s note

All claims expressed in this article are solely those of the authors and do not necessarily represent those of their affiliated organizations, or those of the publisher, the editors and the reviewers. Any product that may be evaluated in this article, or claim that may be made by its manufacturer, is not guaranteed or endorsed by the publisher.
